# Association of human papillomavirus vaccination with cervical cancer screening: A systematic review and meta-analysis

**DOI:** 10.1097/MD.0000000000029329

**Published:** 2022-07-15

**Authors:** Paddy Ssentongo, Jennifer S. McCall-Hosenfeld, William A. Calo, Jennifer Moss, Eugene J. Lengerich, Vernon M. Chinchilli, Djibril M. Ba

**Affiliations:** a Department of Public Health Sciences, Penn State College of Medicine, Hershey, PA; b College of Engineering Science and Mechanics, The Pennsylvania State University, State College, PA; c Department of Medicine, Penn State College of Medicine, Hershey, PA; d Department of Family and Community Medicine, Penn State College of Medicine, Hershey, PA.

**Keywords:** cervical cancer screening, human papillomavirus vaccine, prevention

## Abstract

**Introduction:**

Prophylactic vaccination and routine screening are effective at preventing most cases of cervical cancer. Globally, cervical cancer is the fourth most frequently diagnosed cancer among women. The aim of this study was to investigate the association between human papillomavirus virus (HPV) vaccination (1, 2, or 3 doses) and cervical cancer screening.

**Methods:**

PubMed (MEDLINE), Scopus, Web of Science, and Cochrane Library electronic databases were systematically searched from July 1, 2006, up to September 30, 2021. We pooled estimates using random-effects models. Heterogeneity between studies was quantified using Cochran *Q* test and *I^2^* statistics. In total, 12 studies involving 2.4 million individuals were included in the meta-analysis.

**Results:**

In the adjusted estimates, uptake of HPV vaccination was associated with increased cervical cancer screening (pooled relative risk [RR]: 1.35; 95% confidence interval [CI]: 1.21, 1.50; n = 12). Between-study heterogeneity was large (*I^2^* = 99%). Compared to unvaccinated, those who received 3 doses of HPV vaccine had the highest uptake of cervical cancer screening (RR: 1.85; 95% CI: 1.58, 2.17), followed by those who received 2 doses (RR: 1.34; 95% CI: 1.21, 1.47). No statistically significant association with screening was found for those who received a single dose of the HPV vaccine.

**Conclusion:**

In this meta-analysis, uptake of HPV vaccination was associated with higher cervical cancer screening. It is plausible that vaccinated individuals are more likely to engage in preventive health behaviors. Healthcare providers should remind patients to continue with routine screening for cervical cancer regardless of their HPV vaccine status since vaccination does not protect against all HPV types.

## 1. Introduction

Cervical cancer is a significant public health problem and one of the most preventable types of cancer impacting women worldwide. A recent study using data from the 2020 Global Cancer Observatory (GLOBOCAN) found that cervical cancer was the fourth most frequently diagnosed cancer and the fourth leading cause of cancer death among women worldwide, with >600,000 new cases and 340,000 deaths per year.^[1]^

Human papillomavirus (HPV) is the most common sexually transmitted infection (STI) and a known causal agent of cervical cancer.^[2]^ Sexually active individuals are at a greater risk of becoming infected with HPV than nonsexually active individuals.^[3]^

Effective cervical cancer control measures include HPV vaccination, which is considered primary prevention of cervical cancer, whereas routine cervical cancer screening is an essential secondary prevention strategy.^[4]^ HPV vaccines protect against high-risk HPV strains such as 16 and 18, which cause 70% of cervical cancers and precancerous cervical lesions globally and 90% of genital warts.^[5,6]^ Vaccination with the HPV vaccine does not eliminate the need for routine cervical cancer screening.^[7]^ However, HPV vaccination does not always occur at the recommended ages of 11 to 12 years.^[8,9]^ In addition, individuals who received the vaccine do not always follow the recommended dosing schedule, which may compromise vaccine protection.^[7,10]^

Cervical cancer screening detects precancerous lesions such as abnormal changes in the epithelial cells of the cervix. Prompt treatment of these lesions reduces the risk of cervical cancer.^[11]^ Since the introduction of the Papanicolaou (Pap) test for cervical cancer screening in the 1950s, the number of new cases and deaths due to cervical cancer has decreased gradually by >80% globally.^[12,13]^ It is crucial for individuals with a cervix to undergo routine cervical cancer screening regardless of HPV vaccination status.

Previous studies that have examined the associations between HPV vaccination status and cervical cancer screening have generated mixed results. It is possible that individuals who are vaccinated may be more likely to get screened if they are empowered to manage their cervical health^[7]^; however, it is also possible that patients who are vaccinated may be less likely to get screened if they (incorrectly) perceive that vaccination has provided them complete protection against cervical cancer.^[14]^ To the best of our knowledge, the association between HPV vaccination and cervical cancer screening has not been systematically assessed. To address this gap in the literature, we conducted a comprehensive systematic review and meta-analysis to examine the association of HPV vaccination with cervical cancer screening. Delineating the association between HPV vaccination with cervical cancer screening will help to stratify populations into those who need public health awareness messages on cancer prevention and those that need reinforcement. Although the current cervical cancer screening guidelines do not vary by HPV vaccination status,^[15]^ it is not clear if women’s cervical cancer screening behaviors would be affected by their perceived risk of cervical cancer after initiation of HPV vaccine. The findings from this meta-analysis will contribute to better understanding of cervical cancer screening behaviors in the vaccine era.

## 2. Methods

### 2.1. Identification of studies

**Figure 1.**
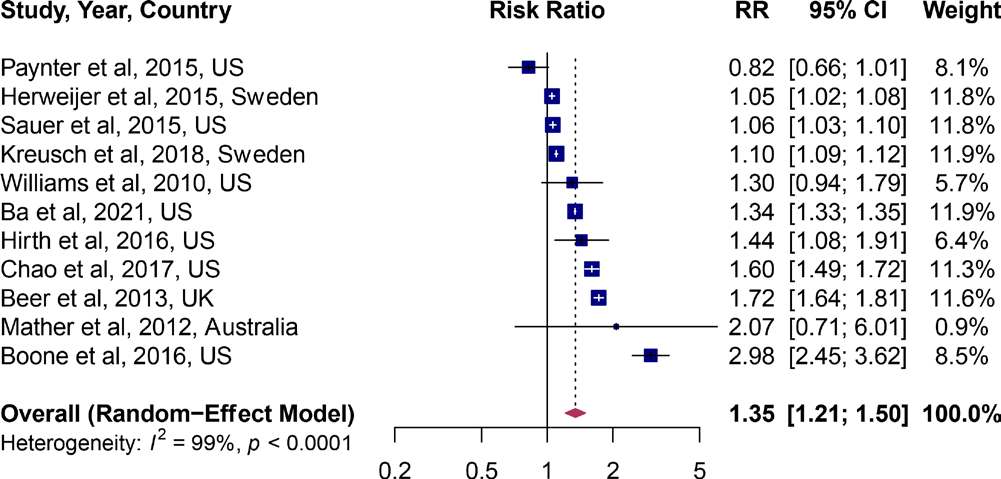
Forest plot of the overall pooled adjusted effect estimate.

This systematic review and meta-analysis used aggregated data without patient identifier. Therefore, ethical approval was not necessary. The reporting of the present study followed the PRISMA (Preferred Reporting Items for Systematic Reviews and Meta-Analyses) guidelines^[16]^ to select relevant published studies for inclusion in this meta-analysis. We conducted a systematic literature search in PubMed (MEDLINE), Scopus, Web of Science, and Cochrane Library databases to identify relevant studies on the association between HPV vaccine uptake and cervical cancer screening published from July 1, 2006, up to September 30, 2021. We restricted our analysis to this timeframe because the first HPV vaccine was licensed in June 2006 for females aged 9 to 26 in the United States.^[17]^ The following keywords were used: “Human Papillomavirus Vaccination” OR “Human Papillomavirus Vaccines” OR “HPV Vaccines” OR “HPV Vaccination” AND “Cervical Cancer Screening.” No language restriction was imposed. The search process is showed in Figure 1, Supplemental Digital Content, http://links.lww.com/MD/G878.

### 2.2. Eligibility criteria

Studies were included in the meta-analysis if they met the following criteria: use an observational study design (cohort or case-control or cross-sectional study designs) or randomized controlled trials; receipt of HPV vaccination as the primary exposure of interest; cervical cancer screening as the outcome of interest; and reported associations in the form of odds ratios (ORs), relative risks (RRs), hazard ratios (HRs), or incidence rate ratio (IRRs) with corresponding 95% confidence interval (CIs).

### 2.3. Data extraction

The data extraction was done by 2 authors independently (D.M.B. and P.S.). Disagreements between D.M.B. and P.S. were resolved by discussion with an available third coauthor to reach an agreement. To evaluate the risk of bias, we used the Newcastle–Ottawa Scale, which consists of domains related to the selection of study groups, the comparability of the groups, and the ascertainment of the outcome of interest.^[18]^ Scores of 8 to the maximum score of 9 were defined as high quality; scores of 5 to 7 were defined as moderate quality and scores of 1 to 4 were defined as low quality. The following data were extracted from each published study: the first author’s name, the year of publication, study period, sample size, outcome assessment, country of study, study design, mean age of participants, the number of cases, reported ORs, RRs, HRs, or IRRs with 95% CIs, duration of follow-up for cohort studies, and the covariates used in the multivariable adjusted models. We extracted 2 types of effect estimates from each study. The unadjusted estimates and the adjusted estimates from multivariable models.

### 2.4. Statistical analysis

As done in a previous study,^[19]^ we combined the crude and adjusted ORs, RRs, HRs, or IRRs as the measures of the association between HPV vaccination and cervical cancer screening. To facilitate the meta-analysis, we used RRs as common risk estimates for all included studies. Two studies reported HPV vaccination and cervical cancer screening for first and second doses^[20]^ and by year of birth^[21]^; in this situation, we combined the effect estimates using a random-effects model to get overall estimates between HPV vaccination and cervical cancer screening within each study.

All reported effect estimates were log-transformed to normalize the distributions. We pooled the effect estimates data from each study, weighted by the inverse of their variances. The *metagen* function from the *R* package meta, which provides the inverse variance method for meta-analysis, was used to calculate the overall effect estimates using random-effects models with the DerSimonian and Laird method.^[22]^ Forest plots were used to graphically display each study effect estimate and overall estimates. Heterogeneity between studies was quantified using Cochran *Q* test and *I^2^* statistics expressed as a proportion (%).^[23]^ The level of significance of heterogeneity was determined with a *P*-value <.05. To explore the potential source of heterogeneity, subgroup and meta-regression analyses were conducted. First, we tested whether the study design affected the outcome of the pooled result. Studies were pooled by cohort versus cross-sectional design. Next, we conducted subgroup analysis by the World Health Organization (WHO) region and the quality of the study. The study year was regressed on the outcome of interest.

Additionally, we conducted an influence sensitivity analysis (Leave-One-Out approach) by removing and replacing 1 individual study at a time to assess the effect of the excluded study on the overall effect estimates. Last, we used Egger and Begg tests and funnel plot asymmetry to examine potential publication bias.^[24,25]^

## 3. Results

### 3.1. Study characteristics

Our first search from July 1, 2006, up to September 30, 2021, produced 2105 potential articles from PubMed, 1949 from Web of Science, Scopus 1042, and 144 from Cochrane library, of which 5228 were excluded (see Fig. 1, Supplemental Digital Content, http://links.lww.com/MD/G878, which shows the PRISMA 2020 Flow Diagram). Twelve studies met our inclusion criteria.^[7,20,21,26–34]^ All reported the number of participants, giving a total of 2.4 million individuals. Six studies reported ORs, and 4 studies reported HRs. The IRR and prevalence ratio were reported by 1 study each. Included studies were cohort (n = 9) and cross-sectional (n = 3). Details of the included studies can be found in Table 1. Eight studies were conducted in the United States, 4 were conducted in Europe (i.e., the United Kingdom [n = 1], Denmark [n = 1], and Sweden [n = 2]), and 1 study was conducted in Australia. Of the studies conducted in the United States, 2 reported race and ethnicity frequency. The proportions of Blacks were 15% and 53% in a study by Sauer et al and Boone et al, respectively. Furthermore, 6 studies reported cervical cancer screening rates stratified by the doses of HPV vaccine received.

**Table 1:** Meta-analysis characteristics of included studies reporting HPV vaccination and cervical cancer screening.

Author, year	Period	Country	Quality score	Study design	RR	RR lower CI	RR upper CI	Vaccinated (n)	Unvaccinated (n)	Statistics
Ba et al^[26]^	2006–2016	US	9	Cohort	1.34	1.33	1.35	190,982	763,928	GEE
Kreusch et al^[30]^	2006–2012	Sweden	8	Cohort	1.10	1.09	1.12	35,460	225,974	Cox regression
Williams et al^[31]^	2010	US	5	Cross-sectional	1.3	1.0	1.9			Logistic regression
Paynter et al^[27]^	2006–2009	US	7	Cohort	0.82	0.67	1.02	1154	1154	Logistic regression
Sauer et al^[29]^	2008–2013	US	5	Cross-sectional	1.08	1.04	1.11			Predicted marginal model
Beer et al^[32]^	2010–2012	UK	8	Cohort	1.72	1.64	1.81	149,666	15,916	Logistic regression
Chao et al^[7]^	2010–2013	US	8	Cohort	1.60	1.49	1.72	17,485	9867	Logistic regression
Boone et al^[34]^	2006–2009	US	7	Cohort	2.98	2.45	3.61	1123	1123	Cox regression
Herweijer et al^[28]^	2006–2012	Sweden	8	Cohort	1.05	1.02	1.08	4897	624,804	Cox regression
Mather et al^[33]^	2011	Australia	6	Cross-sectional	2.07	0.71	5.98	119	74	Logistic regression
Baldur-Felskov et al^[21]^	2006–2012	Denmark	7	Cohort				247,313	151,931	Cox regression
Hirth et al^[20]^	2006–2009	US	7	Cohort	1.44	1.09	1.92			Logistic regression

Missing vaccination information for Williams et al, Sauer et al, and Hirth et al. A study by Baldur-Felskov was added only in the crude pooled estimates. Methodological quality scores are explained in the methods section.

GEE = generalized estimating equation, RR = risk ratio.

The average quality of the studies was moderate, as measured by the Newcastle–Ottawa Scale.

### 3.2. Association of HPV vaccination and subsequent cervical cancer screening

**Figure 2.**
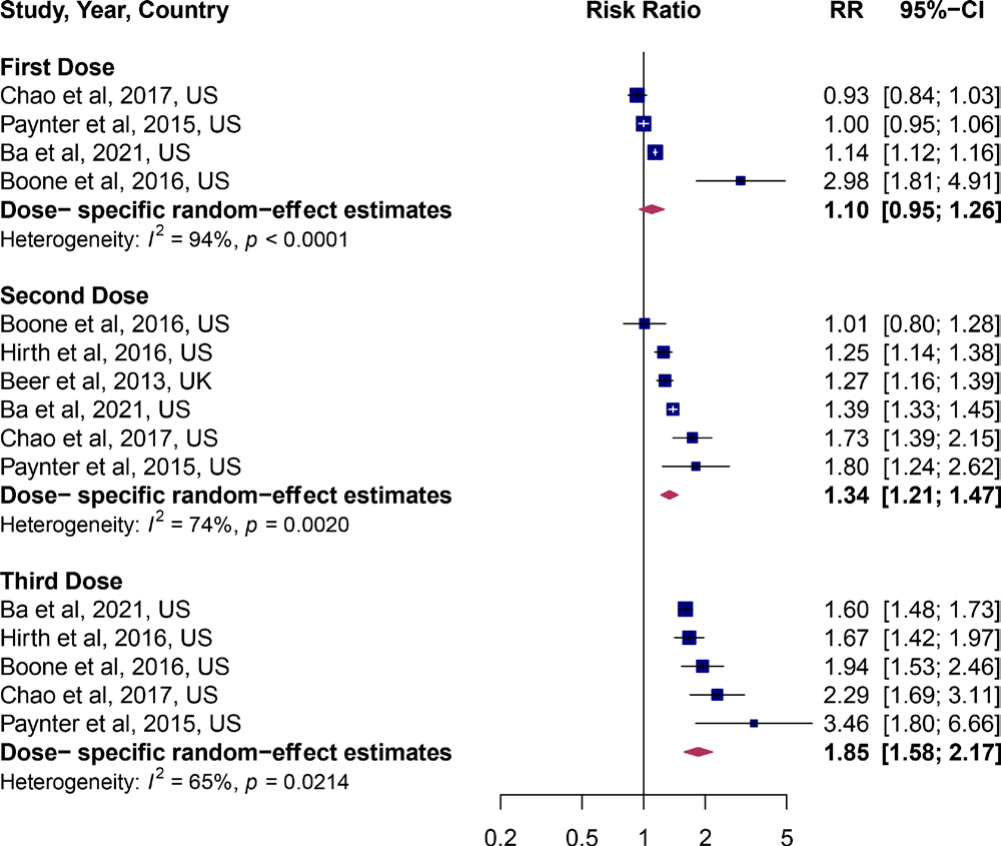
Forest plot of studies stratified by HPV vaccine doses. HPV = human papillomavirus virus.

In the adjusted estimates, HPV vaccination was a significant predictor of uptake of cervical cancer screening (pooled RR: 1.35; 95% CI: 1.21, 1.50), indicating a 35% increased uptake of cervical cancer screening. However, a large amount of heterogeneity between studies was apparent, *I^2^* = 99% (Fig. 1). In the unadjusted estimates (univariate model), HPV vaccination was associated with a 50% higher rate of cervical cancer screening (RR: 1.50; 95% CI: 1.31, 1.71) (see Fig. 2, Supplemental Digital Content, http://links.lww.com/MD/G878, which shows unadjusted estimates). To assess if the dose of the HPV vaccine influenced the observed association, we pooled the estimates by the dose of vaccine received. A dose–response relationship between the number of vaccines received and the uptake of cervical cancer screening was observed. Compared to no vaccine, the uptake of cervical cancer screening was equal in the individuals who received 1 dose (RR: 1.10; 95% CI: 0.95, 1.26, Fig. 2), higher among individuals who received 2 doses (RR: 1.34; 95% CI: 1.21, 1.47, Fig. 2), and highest in individuals who received 3 doses (RR: 1.85; 95% CI: 1.58, 2.17, Fig. 2). Between-study heterogeneity (*I^2^*) decreased from 94% in the first dose, 74% for 2 doses, and 65% for 3 doses.

### 3.3. Subgroup analyses

**Figure 3.**
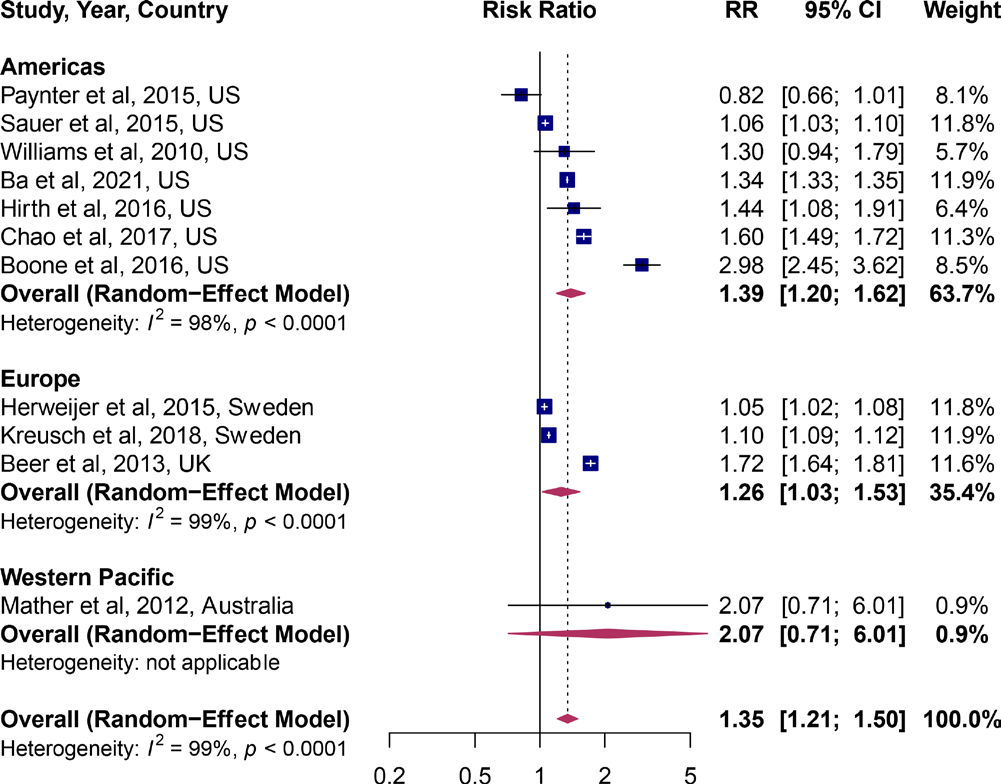
Forest plot of studies stratified by WHO regions. WHO = World Health Organization.

Studies were stratified into subgroups based on their characteristics. Cohort studies showed significant benefit (see Fig. 3, Supplemental Digital Content, http://links.lww.com/MD/G878, which shows subgroup analysis by study design); however, in cross-sectional studies, the beneficial effect did not reach statistical significance.

Next, we investigated the role of the study population in explaining the observed estimates. Studies were assigned to the WHO regions in which they were conducted. In 2 regions – the Americas (United States) and Europe – HPV vaccines had a significant positive association with the receipt of cervical cancer screening (Fig. 3). The most significant effect was among the US studies which included 7 studies, followed by Europe, which included 3 studies.

**Figure 4.**
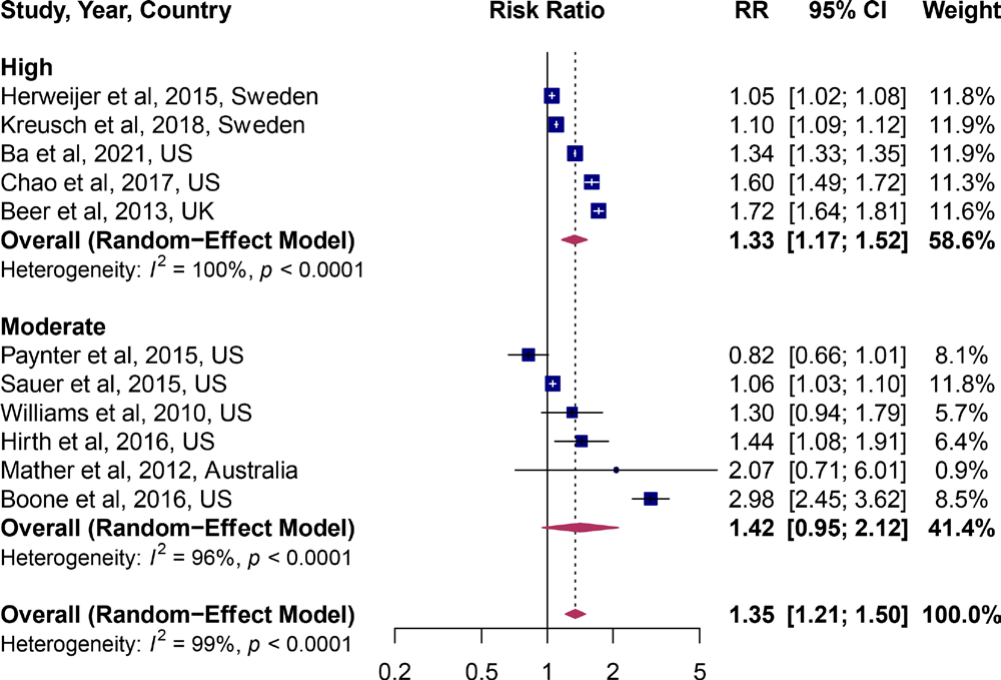
Forest plot of studies stratified by quality score.

Then, we tested whether the study quality affected the outcome of the pooled result. High quality studies demonstrated a statistically significant beneficial effect (pooled RR: 1.33; 95% CI: 1.17, 1.52; n = 5, Fig. 4), while the moderate quality score did not (pooled RR: 1.42; 95% CI: 0.96, 2.11; n = 6).

Last, we tested the impact of the study period on the effect estimates. We chose the median year and used this for analysis. As shown in (see Fig. 4, Supplemental Digital Content, http://links.lww.com/MD/G878, which shows meta-regression analysis), study time frame did not affect the effect estimates (*P* = .25).

### 3.4. Sensitivity analysis and publication bias

Visual inspection of a funnel plot of the included studies (see Fig. 5, Supplemental Digital Content, http://links.lww.com/MD/G878, which shows funnel plot) did not indicate a strong effect of publication bias. Both Egger and Begg tests were nonsignificant. In the outlier analysis, no substantial outlier and influential studies were observed (see Fig. 6, Supplemental Digital Content, http://links.lww.com/MD/G878, which shows influential and outlier analysis). In Duval and Tweedie’s trim and fill test, there were no added studies indicating a lack of adjustment of the funnel plot.

## 4. Discussion

To the best of our knowledge, this is the first meta-analysis to pool available epidemiological studies on the association between HPV vaccination and cervical cancer screening. Our findings indicated that HPV-vaccinated individuals were more likely than unvaccinated to undergo cervical cancer screening. Individuals who received ≥2 doses of HPV vaccines were more likely to be screened for cervical cancer. In contrast, those who received only a single dose of HPV vaccine did not differ from unvaccinated individuals. The observed associations for the adjusted models were similar to the crude effect models, suggesting that unmeasured confounding factors likely had little effect on the results of our meta-analysis.

The association between HPV vaccination uptake and cervical cancer screening could indicate that vaccinated individuals might be more likely to undertake preventive health behaviors and demonstrate higher utilization of primary and secondary medical care. The present findings highlight the potentially significant clinical and public health implications in the prevention of cervical cancer. HPV vaccination is an effective method for primary prevention for most high-risk HPV types associated with cervical cancer. In contrast, cervical cancer screening is secondary prevention to detect changes in the cervix cells or tissue.^[4]^

There are many oncogenic HPV types, and the HPV vaccine does not protect against all HPV types. Thus, not all cervical cancer cases will be prevented by the HPV vaccine. Eligible individuals should continue with routine screening for cervical cancer as recommended regardless of their vaccination status.^[35]^

It is plausible that the observed association between HPV vaccination and cervical cancer screening is driven by cervical cancer knowledge and awareness.^[26,36]^ In addition, the encounters between healthcare providers and women during HPV vaccination may have been an educational opportunity to accentuate the message about continuing cervical cancer screening.^[7]^ Other facilitators of healthcare access, such as health literacy, insurance status, and transportation, may also play important roles in explaining the relationship between vaccination and screening.^[26,29,36,37]^ Healthcare providers play a significant role in advising women to be screened for cervical cancer regardless of their HPV vaccination status during any clinical interactions.

Our meta-analysis has several strengths. In addition to being the first comprehensive meta-analysis on this topic, a significant strength of this work is our use of sensitivity tests and tests for publication bias to attest to the robustness of our data.

Nevertheless, our study has some limitations and the findings should be interpreted with caution. Combining studies from different populations and backgrounds may have resulted in high heterogeneity observed in the current meta-analysis. Nevertheless, subgroup analysis by geographic regions was conducted to tease out potential differences. Additionally, Africa and Asia were not represented, hence the findings of the current analysis many not be generalizable to all WHO regions of the world.

## 5. Conclusion

This systematic review and meta-analysis of observational studies found an association between HPV vaccination with higher cervical cancer screening rates. Individuals who received 2 or more doses of HPV vaccines were more likely to be screened for cervical cancer. The findings of this study have significant public health and clinical implications in preventing cervical cancer and premature deaths. The association of HPV vaccination with cervical cancer screening indicates that vaccinated individuals are more likely to engage in preventive health behaviors. Thus, it is important for healthcare providers to remind patients to continue with routine screening for cervical cancer regardless of their HPV vaccine status since vaccination does not protect against all HPV types.

## Author contributions

Designed research (project conception, development of overall research plan, and study oversight), analyzed data, and wrote the first draft of the manuscript: P.S. and D.M.B.

Performed statistical analysis: P.S.

Review and editing: P.S., J.H., W.C., J.M., E.J.L., V.M.C., and D.M.B.

All authors have read and approved the final manuscript.

## Supplementary Material


