# Supplementary material for: Association of human papillomavirus vaccination with cervical cancer screening: A systematic review and meta-analysis
Source: Medicine (Baltimore). 2022 Jul 15;101(28):e29329. doi: 10.1097/MD.0000000000029329 (PMC11132352; doi:10.1097/MD.0000000000029329)
Supplement: Supplementary file 1 [file medi-101-e29329-s001.pdf]

Supplement to

Association of Human Papillomavirus Vaccination with Cervical Cancer Screening: A Systematic Review and Meta-Analysis of Observational Studies

Paddy Ssentongo, MD, PhD, MPH[1,2], Jennifer S. McCall-Hosenfeld, MD, MSc[3,1], Vernon M. Chinchilli, PhD[1], Eugene J. Lengerich, V.M.D., M.S.[1], William Calo, PhD, JD, MPH[1], Djibril M. Ba, PhD, MPH[1]

[1]Department of Public Health Sciences, Penn State College of Medicine, Hershey, PA, USA

[2]College of Engineering Science and Mechanics, The Pennsylvania State University, State College, PA, USA

[3]Department of Medicine, Penn State College of Medicine, Hershey, PA, USA

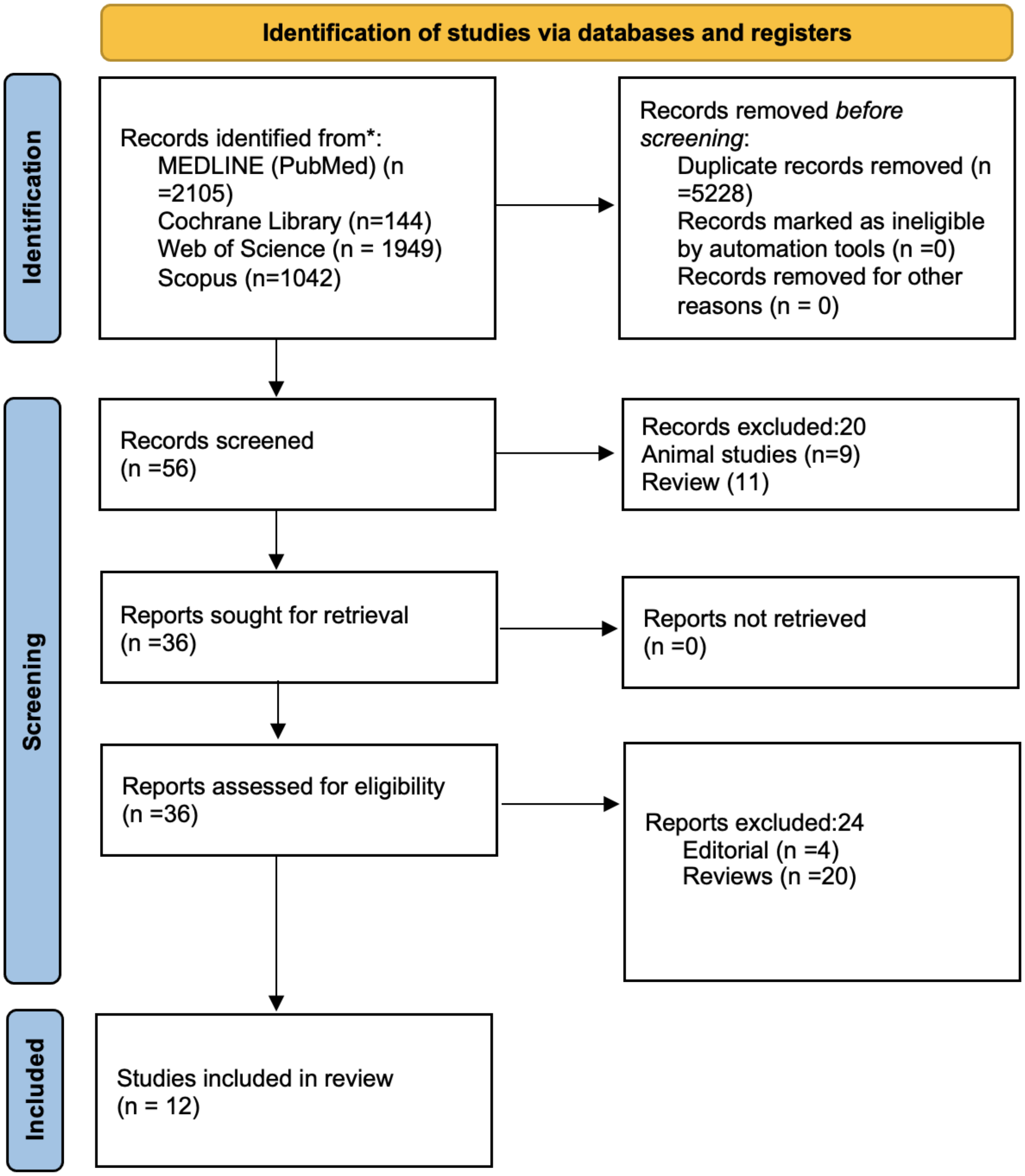

Supplemental Figure 1: PRISMA flow diagram showing study selection process.

Supplement to

Association of Human Papillomavirus Vaccination with Cervical Cancer Screening: A Systematic Review and Meta-Analysis of Observational Studies

Paddy Ssentongo, MD, PhD, MPH[1,2], Jennifer S. McCall-Hosenfeld, MD, MSc[3,1], Vernon M. Chinchilli, PhD[1], Eugene J. Lengerich, V.M.D., M.S.[1], William Calo, PhD, JD, MPH[1], Djibril M. Ba, PhD, MPH[1]

[1]Department of Public Health Sciences, Penn State College of Medicine, Hershey, PA, USA

[2]College of Engineering Science and Mechanics, The Pennsylvania State University, State College, PA, USA

[3]Department of Medicine, Penn State College of Medicine, Hershey, PA, USA

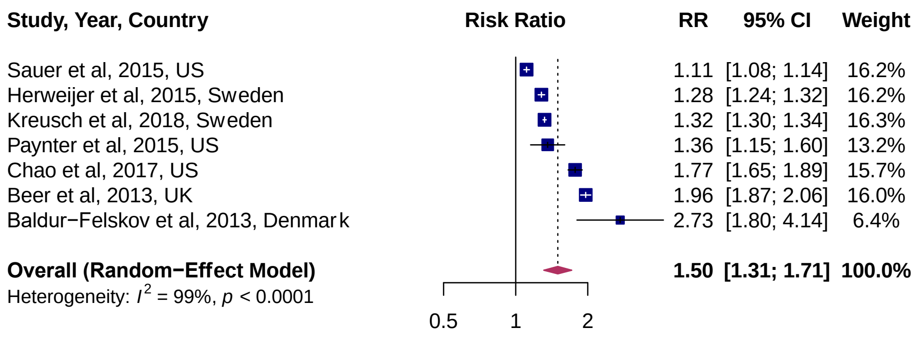

Supplemental Figure 2: Forest plot of the overall pooled crude effect estimate.

Supplement to

Association of Human Papillomavirus Vaccination with Cervical Cancer Screening: A Systematic Review and Meta-Analysis of Observational Studies

Paddy Ssentongo, MD, PhD, MPH[1,2], Jennifer S. McCall-Hosenfeld, MD, MSc[3,1], Vernon M. Chinchilli, PhD[1], Eugene J. Lengerich, V.M.D., M.S.[1], William Calo, PhD, JD, MPH[1], Djibril M. Ba, PhD, MPH[1]

[1]Department of Public Health Sciences, Penn State College of Medicine, Hershey, PA, USA

[2]College of Engineering Science and Mechanics, The Pennsylvania State University, State College, PA, USA

[3]Department of Medicine, Penn State College of Medicine, Hershey, PA, USA

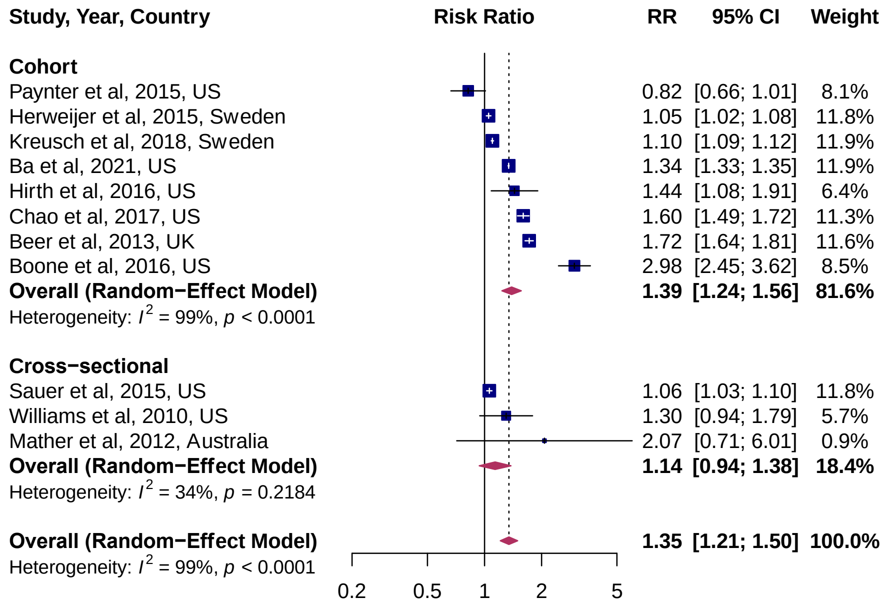

Supplemental Figure 3: Forest plot of studies stratified by study design.

Supplement to

Association of Human Papillomavirus Vaccination with Cervical Cancer Screening: A Systematic Review and Meta-Analysis of Observational Studies

Paddy Ssentongo, MD, PhD, MPH[1,2], Jennifer S. McCall-Hosenfeld, MD, MSc[3,1], Vernon M. Chinchilli, PhD[1], Eugene J. Lengerich, V.M.D., M.S.[1], William Calo, PhD, JD, MPH[1], Djibril M. Ba, PhD, MPH[1]

[1]Department of Public Health Sciences, Penn State College of Medicine, Hershey, PA, USA

[2]College of Engineering Science and Mechanics, The Pennsylvania State University, State College, PA, USA

[3]Department of Medicine, Penn State College of Medicine, Hershey, PA, USA

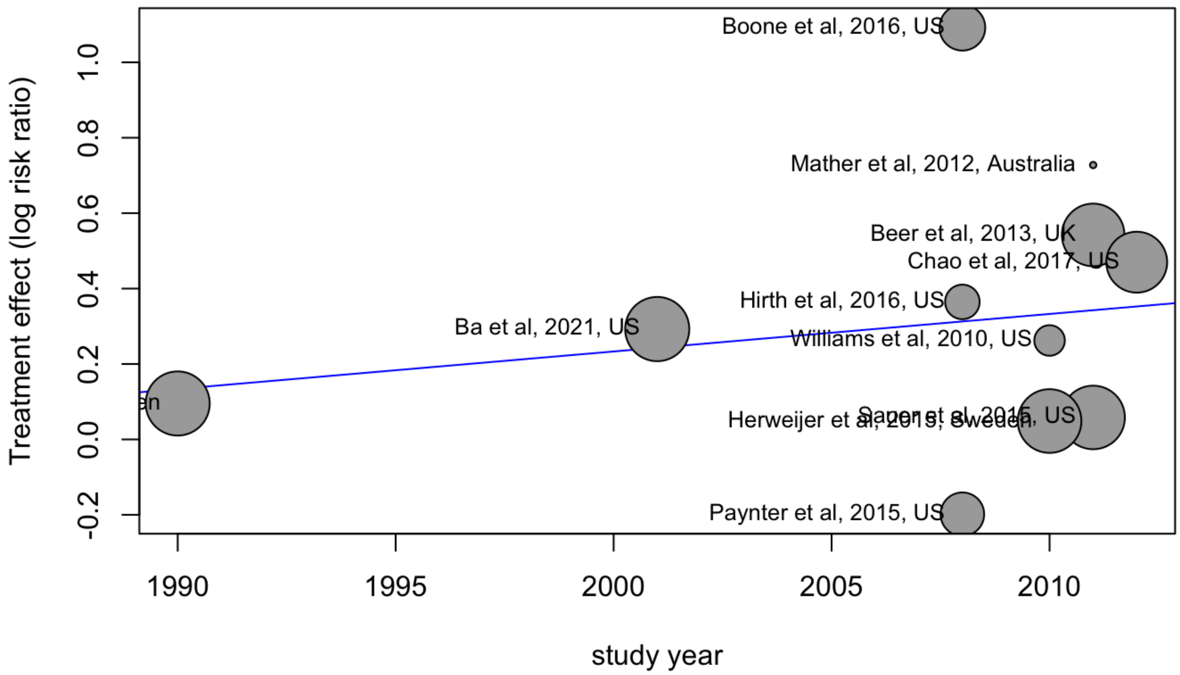

Supplemental Figure 4: Meta-regression.

Supplement to

Association of Human Papillomavirus Vaccination with Cervical Cancer Screening: A Systematic Review and Meta-Analysis of Observational Studies

Paddy Ssentongo, MD, PhD, MPH[1,2], Jennifer S. McCall-Hosenfeld, MD, MSc[3,1], Vernon M. Chinchilli, PhD[1], Eugene J. Lengerich, V.M.D., M.S.[1], William Calo, PhD, JD, MPH[1], Djibril M. Ba, PhD, MPH[1]

[1]Department of Public Health Sciences, Penn State College of Medicine, Hershey, PA, USA

[2]College of Engineering Science and Mechanics, The Pennsylvania State University, State College, PA, USA

[3]Department of Medicine, Penn State College of Medicine, Hershey, PA, USA

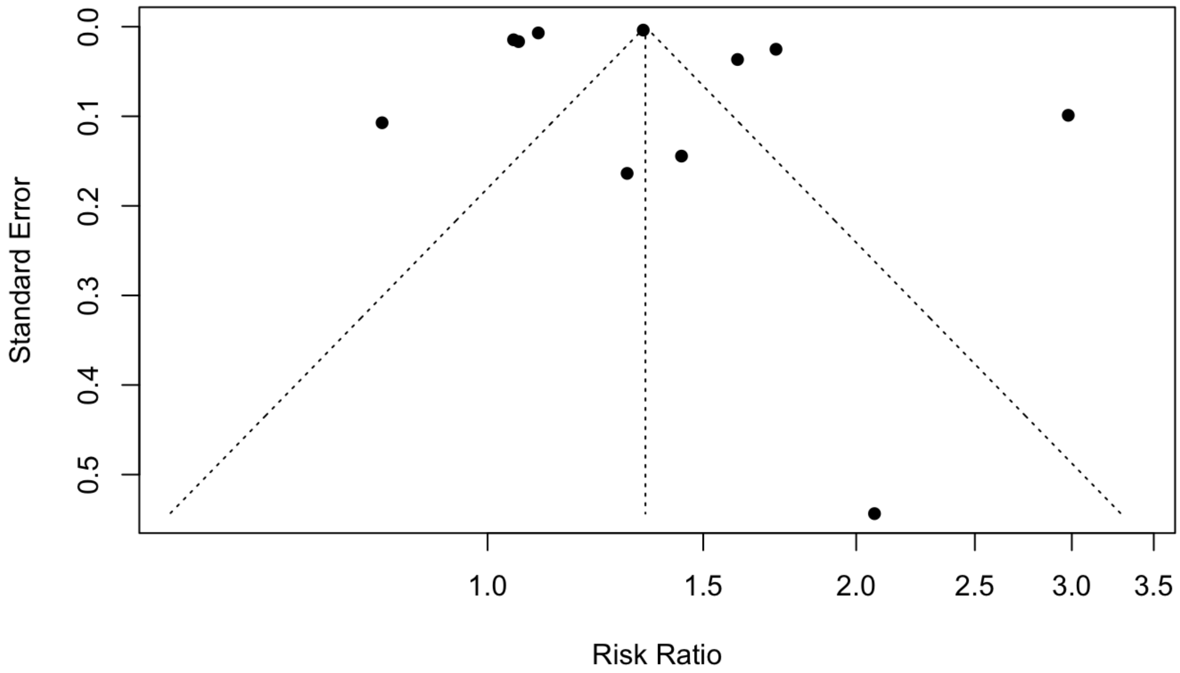

Supplemental Figure 5: Funnel plot

Supplement to

Association of Human Papillomavirus Vaccination with Cervical Cancer Screening: A Systematic Review and Meta-Analysis of Observational Studies

Paddy Ssentongo, MD, PhD, MPH[1,2], Jennifer S. McCall-Hosenfeld, MD, MSc[3,1], Vernon M. Chinchilli, PhD[1], Eugene J. Lengerich, V.M.D., M.S.[1], William Calo, PhD, JD, MPH[1], Djibril M. Ba, PhD, MPH[1]

[1]Department of Public Health Sciences, Penn State College of Medicine, Hershey, PA, USA

[2]College of Engineering Science and Mechanics, The Pennsylvania State University, State College, PA, USA

[3]Department of Medicine, Penn State College of Medicine, Hershey, PA, USA

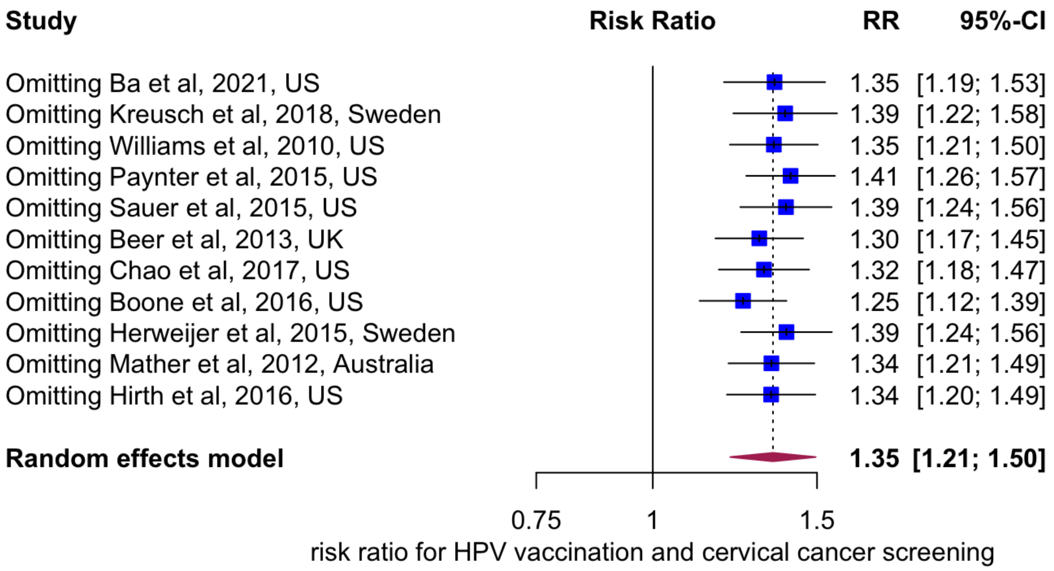

Supplemental Figure 6: Influential and outlier analysis.
